# Fecal DNA metabarcoding reveals seasonal and annual variation in willow ptarmigan diet

**DOI:** 10.1098/rsos.231518

**Published:** 2024-02-28

**Authors:** Elise W. Ingvaldsen, Jan E. Østnes, Oddmund Kleven, Marie Davey, Frode Fossøy, Erlend B. Nilsen

**Affiliations:** ^1^ Faculty of Biosciences and Aquaculture, Nord University, 7713 Steinkjer, Norway; ^2^ Norwegian Institute for Nature Research, 7485 Trondheim, Norway

**Keywords:** alpine bird, *Lagopus lagopus*, dietary study, environmental DNA, remote sensing

## Abstract

Understanding spatio-temporal variation in the diet of alpine herbivores is important to predict how a changing climate will affect these species in the future. We examined the spatio-temporal variation in willow ptarmigan (*Lagopus l. lagopus*) diet using DNA metabarcoding of fecal pellets sampled from winter to early summer over three consecutive years. Furthermore, we assessed how snow cover and vegetation phenology affected diet variation. We also investigated sex differences in diet composition. We identified 18 important diet taxa and the genera *Betula, Vaccinium* and *Empetrum* occurred most frequently. Diet composition and richness varied within and between years. Seasonally, there was a shift from a narrow winter diet dominated by trees and dwarf shrubs to a broader spring diet with more nutritious field vegetation. This seasonal progression differed among years. The temporal variation in diet was better explained by day of year than by snow cover and vegetation phenology. Females had a more diverse diet than males, but there were no sex differences in diet composition. Our results demonstrate that metabarcoding of fecal samples provides the opportunity to assess factors affecting diet composition of species in alpine ecosystems in the context of a changing climate.

## 1. Introduction

For herbivorous species in alpine ecosystems, snow cover and a short plant growing season make access to nutritious food during winter and spring a potentially limiting factor [1]. In the alpine ecosystems in Fennoscandia, climate change is expected to result in an earlier onset of spring and a longer growing season [2]. Changes in plant phenology caused by a warmer climate are well documented [3], and the most pronounced are seen in alpine areas where duration of snow cover affects plant growth and vegetation composition [4]. For herbivorous resident bird species living in such environments, increased temperature and earlier snow melt may lead to an earlier access to green nutrient-rich plant material which might be beneficial in terms of increased reproductive output [5]. Knowledge about spatio-temporal variation in the diet of alpine species might therefore help us understand species interactions and ecosystem functions under these novel and changing conditions [6].

Willow ptarmigan (*Lagopus l. lagopus*) is a ground foraging herbivorous bird with a northern circumpolar distribution [7]. In Fennoscandia, willow ptarmigan inhabit the subalpine to alpine bioclimatic zone, from mountain birch forest to alpine heath habitats [8]. It is a keystone prey species in the alpine ecosystem [9,10]. As a non-migratory species, their life history is adapted to a life in a seasonal ecosystem. During winter, their habitats are mainly covered by snow, and snow depth strongly affects foraging behaviour [11]. They use plants that are available above the snow cover and can survive the winter on a very lean plant diet [12]. Previous studies suggest that during winter, willow ptarmigan feed almost exclusively on shoots and catkins of mountain birch (*Betula pubescens*), and shoots and flower buds of willows (*Salix* spp.) and bilberry (*Vaccinium myrtillus*) stems [13].

In spring, when the first snowless spots appear, willow ptarmigan switch to feeding on field layer vegetation [14]. This switch causes variation in diet richness and composition among seasons [15]. Nutritious field layer vegetation is the primary food resource during the egg-laying period, and diet composition significantly affects breeding success and survival of ptarmigans [13,15]. Plant phenology and vegetation composition in willow ptarmigan habitats are tightly linked to the timing and distribution of snow [1], and spring snow cover determines which plant species are available as food sources [16]. Because snow cover and plant phenology typically display annual spatial and temporal variation, inter-annual variation in the types of plants available for willow ptarmigan at different times of the year are expected. In years with early snowmelt, they can benefit from a longer period of high-quality food resources, and this affects the condition of the birds [17]. A warmer climate that provides earlier access to fresh nutritious plants can thus potentially have a positive effect for willow ptarmigan, at least on a short-term basis [10].

Male and female willow ptarmigans have different energy requirements due to different behaviour during the breeding season. Males start their territorial calling and habitat defense in March [18]. During this period, males eat less and lose weight [19]. At the same time, the females must build up their body reserves to prepare for egg-laying and brooding, and this difference between the sexes is known to lead to sex-specific foraging behaviours and habitat selectivity [20–22]. Despite these behavioural differences, several studies have not been able to demonstrate any sex differences in the diet composition of ptarmigan during the winter and spring seasons [14–16].

Here, we use DNA metabarcoding of fecal pellets to investigate the diet composition and richness of willow ptarmigan in central Norway during the transition period from winter to early summer in three consecutive years. Based on previous research, we predict that (i) willow ptarmigan has a narrow winter diet dominated by mountain birch but shifts to a broader spring diet with increasing elements of nutritious field vegetation as the spring progresses. Further, (ii) we predict that this increase in nutritious field vegetation and shift in diet components varies between years and can be explained by snow cover and vegetation phenology in the study area. Finally, we predict (iii) that the diet composition of males and females differ.

## 2. Material and methods

### 2.1. Study site

**Figure 1.**
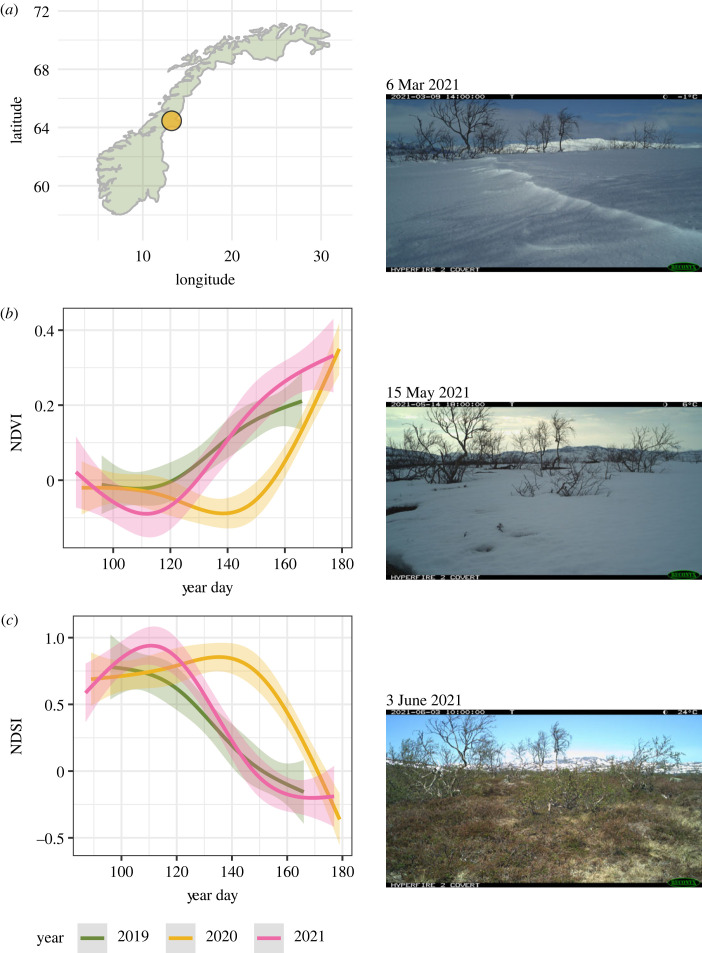
Left side: the location of the study area in central Norway (*a*). Estimated relationship (solid line) between (*b*) predicted NDVI-values and day of the year and (*c*) predicted NDSI-values and day of the year. The shaded ribbons represent 95% confidence interval. Right side: images taken by one of the game cameras in the study area, illustrating the development of snowmelt and green biomass throughout the spring in 2021.

Data were collected at Lifjellet (64°27 N, 13°14 E) in central Norway (figure 1*a*), during the transition from winter to early summer over three consecutive years (2019–2021). Lifjellet is situated in the subalpine to alpine bioclimatic zone [23]. The lower parts of the study area are dominated by sparse forest with Norway spruce (*Picea abies*), and Scots pine (*Pinus sylvestris*), interspersed with mountain birch and *Salix* spp. At medium elevations, there are more open mountain vegetation with dwarf birch (*Betula nana*) and scattered patches of a few other tree species. Large parts of these areas are bogs covered by grasses and sedges, interspersed with drier areas with ericaceous dwarf shrubs. At the highest elevations, the landscape alternates between exposed ridges, leesides with dwarf shrubs, snowbeds and shallow bogs. Annual average precipitation in the area is approx. 675 mm yr^−1^ [24]. Temperatures span from an annual average of −10°C in January to +12°C in July [24]. In winter, snow depth is 1–3 m, and snow cover typically persists from early October to late May [25].

### 2.2. Fecal collection

Fecal pellets from ptarmigan were collected weekly from March to June in the years 2019–2021. Willow ptarmigan inhabit areas that overlap with the sympatric rock ptarmigan (*Lagopus muta*), and it is difficult to distinguish between pellets of the two species based on visual appearance. Therefore, species identity was determined based on DNA microsatellite analyses (see below), and only pellets from willow ptarmigan were included in this study. The surveys were carried out by a field team consisting of two persons, within seven predefined sampling plots (100 m × 100 m), located along an altitudinale gradient. The sampling plots were connected by a predefined transect with a total distance of 7 km. Fecal pellets were collected within each of the sampling plots, and opportunistically along the transect. A handheld GPS (Garmin GPSmap 62 s) was used to follow the transect and to identify the boundaries of the squares. Track logs and geographical coordinates for samples collected along the transect were stored. Because DNA degrades as the fecal pellets age, we collected pellets assumed to be no older than 0–3 days based on the following criteria: (1) pellets were always collected from willow ptarmigans observed along the transect and within the squares. (2) Pellets found on or close to fresh tracks were considered as fresh. In addition, (3) we used the appearance of the pellets to evaluate the age. Pellets change colour and texture depending on what the ptarmigan has eaten, but a fresh pellet will have an even colour and be soft in contrast to an older pellet which dries out and becomes greyer on the underside. Single pellets were placed into separate sterile airtight tubes containing silica gel (granulate size 1–3 mm from Chameleon in 2019 and 2020, granulate size 2–5 mm from Real Marine in 2021) for desiccation. To minimize repeated sampling of the same individual on the same day only fecal pellets that were found at least 5 m apart were collected. A maximum of five fecal pellets were collected from each sampling plot at each visit. After collection, the sample tubes were stored under dry and dark conditions at room temperature prior to DNA extraction.

A total of 399 fecal samples were collected (140 in 2019, 187 in 2020 and 72 in 2021, respectively). Owing to financial constraints we had to make a selection, and 141 samples were selected for DNA extraction. We did expect that some samples would not amplify and that some of the samples could originate from rock ptarmigan. Taking this into account, samples from each year were organized into seven equal time periods (blocks). From these blocks, we selected pellets that were assumed to be as fresh as possible, and we selected samples across years and blocks to achieve a balanced design (i.e. equal number of samples from each of the seven blocks and from each year). For interpretation of results and for use in further analyses, the samples were also organized into three different time periods (table 1).

**Table 1.** The final dataset of 99 fecal pellets from willow ptarmigan divided into years and blocks. Block seven was not sampled in 2019. The distribution of the 141 samples originally selected for DNA extraction is shown in parenthesis.

period	winter		late spring		early summer		
	Block 1	Block 2	Block 3	Block 4	Block 5	Block 6	Block 7
	26.03–04.04	09.04–17.04	23.04–03.05	07.05–16.05	21.05–30.05	04.06–14.06	18.06–26.06
2019	6 (8)	6 (9)	5 (7)	7 (7)	3 (7)	1 (8)	-
2020	4 (7)	7 (7)	7 (7)	2 (9)	5 (7)	8 (8)	6 (8)
2021	3 (7)	7 (7)	7 (7)	7 (7)	4 (7)	3 (6)	1 (1)

### 2.3. Genetic analyses

#### 2.3.1. Extraction of DNA

The genetic analyses were carried out by the Centre for Biodiversity Genetics (NINAGEN) at the Norwegian Institute for Nature Research (NINA) in Trondheim, Norway. DNA was extracted from fecal pellets using the MP Biomedicals FastDNA SPIN Kit for Soil (MP Biomedicals, Germany) following the manufacturers protocol.

#### 2.3.2. Microsatellite genotyping and sex determination

All scat samples were genotyped with nine microsatellite markers to determine species and individual identity. An additional marker was included for sex determination. For additional information about the genotyping, see electronic supplementary material.

#### 2.3.3. Diet analyses

To characterize the diet of willow ptarmigan, we used metabarcoding of the ITS2 region of rDNA. The target region was amplified using the ITS2-S2F (5′- 142 ATGCGATACTTGGTGTGAAT-3′) [26] and ITS4 (5′-TCCTCCGCTTATTGATATGC-3′) primers, which amplify a wide variety of flowering plant families, including many previously reported in willow ptarmigan diet (e.g. Betulaceae, Salicaceae, Poaceae, Ericaceae, Empetraceae) [27,28]. PCR was conducted in 25 µl volumes containing: 1X KAPA HiFi HotStart Ready Mix, 0.5 µM of each primer and 2.5 µl of 10 ng µl^−1^ template DNA. The thermal cycling profile was 95°C for 3 min, 95°C for 30 s, 35 cycles of 56°C for 30 s, 72°C for 30 s and a final extension of 10 min at 72°C. IDT for Illumina DNA/RNA UD indices were added to the 5′ and 3′ ends of the amplicons according to the manufacturer's instructions. Amplicons were diluted to 7 ng µl^−1^, and magnetic beads were used to remove fragments under 200 bp and over 600 bp. Amplicons were pooled in equimolar amounts and sequenced on an Illumina NovaSeq 6000 platform at the Norwegian Sequencing Centre (NSC) in Oslo, Norway.

Demultiplexing of the samples and adapter removal was conducted on the Illumina NovaSeq platform. Cuadapt v.1.18 [29] was used to remove the 5′ primer and the 3′ primer and any additional index and adapter bases, requiring a minimum length match of 17 bp with 0.15 expected errors. Quality filtering, error correction, merging, mapping and chimera removal were conducted using the DADA2 v.1.22 package for R [30]. Reads were quality-filtered to remove all sequences with ambiguous bases, with greater than 2 expected errors in both the forward and reverse direction, and length less than 50 bp after truncation at the first instance of a base with a quality score less than 10. Error rates were estimated with enforced monotonicity for forward and reverse sequences, forward and reverse reads were merged with a minimum overlap of 30 bp, and amplicon sequence variants (ASVs) were generated for each sample. Sequence variants that were flagged as chimaeric in more than 90% of the samples were removed from the dataset. Taxonomy was assigned to all non-chimaeric ASVs using the Sintax algorithm [31] with default k-mer and sub-sample size and 100 bootstrapping iterations. Sequences were compared to a custom database of publicly available ITS sequences for Norwegian plant species that was assembled by filtering the publicly available PLANiTS database to include only those members of Streptophyta listed in the Norwegian Biodiversity Information Center's taxonomic database (https://www2.artsdatabanken.no/artsnavn/Contentpages/Eksport.aspx). A minimum of 80% confidence was required for successful assignment at a given taxonomic level. Any ASV that could not be successfully classified to Streptophyta with greater than 80% confidence using the Sintax algorithm was removed from the dataset. All non-chimaeric ASVs were additionally subjected to Megablast comparisons against GenBank to identify and remove non-target ASVs: any ASV with a best BLAST match of greater than 90% identity and greater than 90% coverage to a non-Streptophyta taxon was considered non-target and removed from the dataset. A total of 86 Streptophyta ASVs were identified using the Sintax-Megablast approach described above (electronic supplementary material, table S2), of which 5 (5.8%) were assigned to family level, 21 (24.4%) to genus level and 60 (69.8%) to species level. The ASVs were further grouped into six different functional groups; forbs, trees, graminoids, dwarf shrubs and bryophytes. The Species Map Service for Norway (artskart.artsdatabanken.no) and the expert opinion of a botanist from Nord university (Assoc. Prof. Håkon Holien) was used to identify potential contaminants, i.e. plant species unlikely to occur in the study area (27 of 86 ASVs). To mitigate potential effects of contaminants on the dataset, we excluded all species with equal or lower sequence abundance as the most abundant putative contaminant (0.014%). All other species removed in this operation comprised a very small proportion of the sequences and thus were considered insignificant dietary components. From the 141 fecal pellets selected for DNA analysis, DNA of sufficient quality for species, sex, and individual determination was extracted from 121 samples. Based on the microsatellite data, 22 of these originated from rock ptarmigan and were excluded from further analyses. The final dataset thus consisted of 18 Streptophyta ASVs from 99 willow ptarmigan fecal samples, 50 males and 49 females (table 1). All further analyses were based on these 18 ASVs of which four were assigned to genus level and 14 to species level (table 2). In the final dataset, 19 ptarmigan individuals were represented with more than one data point; the total number of unique ptarmigan individuals was 58, and the median number of samples per individual was 1 (range: 1–12).

**Table 2.** Number of occurrences (N.o.) and frequency of occurrence (FO %) of important dietary components found across the samples from willow ptarmigan (*n* = 99).

family	species	functional groups	N.o.	FO %
Betulaceae	*Betula nana*	trees	95	95.96
Betulaceae	*Betula* sp.	trees	95	95.96
Ericaceae	*Empetrum nigrum*	dwarf shrubs	80	80.81
Ericaceae	*Vaccinium myrtillus*	dwarf shrubs	77	77.78
Ericaceae	*Vaccinium* sp.	dwarf shrubs	45	45.45
Betulaceae	*Betula pubescens*	trees	42	42.42
Ericaceae	*Vaccinium uliginosum*	dwarf shrubs	36	36.36
Ericaceae	*Andromeda polifolia*	dwarf shrubs	30	30.3
Ericaceae	*Calluna vulgaris*	dwarf shrubs	24	24.24
Ericaceae	*Vaccinium vitis-idaea*	dwarf shrubs	19	19.19
Cyperaceae	*Eriophorum* sp.	graminoids	19	19.19
Rosaceae	*Rubus chamaemorus*	forbs	16	16.16
Rosaceae	*Sorbus aucuparia*	trees	9	9.09
Cornaceae	*Chamaepericlymenum suecicum*	forbs	8	8.08
Salicaceae	*Salix* sp.	trees	7	7.07
Orobanchaceae	*Melampyrum pratense*	forbs	7	7.07
Ericaceae	*Oxycoccus microcarpus*	dwarf shrubs	5	5.05
Rosaceae	*Potentilla erecta*	forbs	3	3.03

### 2.4. Data preparation

#### 2.4.1. Index of spring progress and snow cover

To examine how season affects diet, three covariates were used: Julian Date, the Normalized difference vegetation index (NDVI) and the Normalized difference snow index (NDSI). NDVI was used to calculate the expansion of gross primary production in the study area [32]. NDVI and NDSI were constructed from Sentinel 2 satellite imagery using the Google Earth Engine platform. The boundary for image collection was defined by a polygon that represented the study area with a 200 m buffer zone. To filter out clouds, water and shadows from the images, we used the algorithm described by Hollstein *et al*. [33]. NDVI and NDSI values were extracted from 10 000 random points in the study area from the imagery collection. Further processing of the values was carried out using R version 4.1.2 (R Development Team 2021), using the package *xts* [34] to create weekly averages of NDVI and NDSI. Then, we used generalized additive models (implemented in the R-package *mgcv* [35]) to create smoothed time series of NDVI and NDSI, respectively. We used the predictions from these models as measures of NDVI and NDSI for the study area for each sampling date.

In general, snow cover and the arrival of spring varied substantially between the 3 years of data collection (figure 1*b*,*c*). In 2019, snow cover was sparse and with an early snowmelt and greening, whereas in 2020 large parts of the study area were covered by snow until the middle of June. The year 2021 represented an average between the two previous years.

#### 2.4.2. Quantifying the ptarmigan diet

Two indicators are frequently used to quantify dietary data based on fecal metabarcoding: frequency of occurrence (FO) and relative read abundance (RRA) [36]. FO represents the presence/absence of each taxon in each fecal pellet [36], whereas the RRA represents the proportion of identified sequence ­reads for each taxon in a sample [36]. Both indicators have some known potential biases: FO can overestimate the importance of plant taxa consumed only in small amounts, while RRA does not necessarily reflect the true proportion of consumed plant taxa [37]. To reduce biases by reporting only one, we used both dietary metrics. Our diet richness analyses were based on FO, while we used RRA to analyse compositional differences in the diet.

### 2.5. Statistical analyses

As a measure of dietary richness, we calculated the number of species, families, and functional groups per fecal sample (based on the FO data). We used generalized linear models to assess how dietary richness varied in the transition from winter to early summer for males and females, respectively. In total, 19 candidate models were considered (electronic supplementary material, table S3). Because the response variable was under-dispersed, we used a Conway–Maxwell–Poisson distribution, fitted using the function glm.cmp in package mpcmp [38]. To further assess how dietary richness and composition of willow ptarmigan varied across time, we separately modelled the FO and RRA of the three most frequent genera in the willow ptarmigan diet (*Betula*, *Vaccinium* and *Empetrum*, table 2). For each taxa and for both FO and RRA, we constructed and compared ten candidate models (electronic supplementary material, tables S7, S9, S11, S13, S15 and S19). To model FO data, we used a logit link function (assuming binomial error distribution). Four candidate models were excluded due to Hauck–Donner effects (*Vaccinium* FO ∼ jDate × Year, *Empetrum* FO ∼ jDate × Year, *Empetrum* FO ∼ NDVI × Year and *Empetrum* FO ∼ NDSI × Year). Because RRA is a proportion between 0 and 1, we logit-transformed the data prior to analyses [39], and used general linear models assuming Gaussian error distribution. To evaluate the relative support of the candidate models, we used the Akaike Information Criterion corrected for small sample sizes (AICc) [40]. We considered the less complex model within a ΔAICc range of 0–2 as the most parsimonious model and considered models with a ΔAICc value >2 as inconclusive [41].

Because we had repeated observations from some individuals (see chapter above about diet analyses), we initially considered using mixed-effects models to account for potential non-independence across samples. However, initial tests suggested that the amount of residual variation that was accounted for by individuals was in most cases negligible (less than 1%). We therefore used AICc to evaluate the need for including a random term for individual ID. In all but one case the models without a random intercept for individuals were more parsimonious. The only exception was FO for vaccinium, where there were some indications that a random term was appropriate (ΔAICc = 7.73). In this case, we repeated the analyses presented in the main text by including only the first observation for each bird. All mixed-effects models were run using function glmmTMB in package *glmmTMB* [42], with link function and error structure similar to their glm counterparts.

To assess variation in dietary composition among samples from different seasons and years and between males and females, we calculated distance matrixes for the samples based both on the FO and the RRA-data, using the distance function from the R-package *phyloseq* [43]. We calculated Jaccard distance matrix for the FO-data and Bray–Curtis distance matrix for the RRA-data [44]. We performed two separate multivariate analysis of variance (perMANOVA) models with 999 permutations to test for the effect of season, sampling year and sex on the dietary composition using the adonis function in the R-package *vegan* [45]. The assumption of homogeneity of multivariate dispersion was tested using the *betadisper* and *permutest* function in R-package *vegan* with 999 permutations. The homogeneity in variance assumption was met for the predictor variables period (d.f. = 2, *F* = 0.22, *p* = 0.797) and sex (d.f. = 1, *F* = 0.22, *p* = 0.634), but not for year (d.f. = 2, *F* = 4.62, *p* = 0.017) in the FO-models, whereas the homogeneity of variance assumption was met for predictor variables sex (d.f. = 1, *F* = 1.06, *p* = 0.31) and year (d.f. = 2, *F* = 0.87, *p* = 0.43), but not for period (d.f. = 2, *F* = 16.13, *p* = 0.001) when modelling RRA. This suggests that some of the reported effects might be inflated.

All statistical analyses were carried out using R version 4.1.2 (R Development Team 2021).

## 3. Results

### 3.1. Dietary richness and composition

Based on FO data, the most common functional groups in the diet were trees (97%), and dwarf shrubs (89%). Graminoids and forbs were present in 20% and 19% of the samples, respectively. Two families, *Betulaceae* (96%) and *Ericaceae* (88%), were present in most of the samples, represented by the genera *Betula, Empetrum* and *Vaccinium.* The four most frequently occurring ASVs were identified as *Betula nana* (96% of samples), *Betula* sp. (96%) *Empetrum nigrum* (81%) and *Vaccinium myrtillus* (78%).

**Figure 2.**
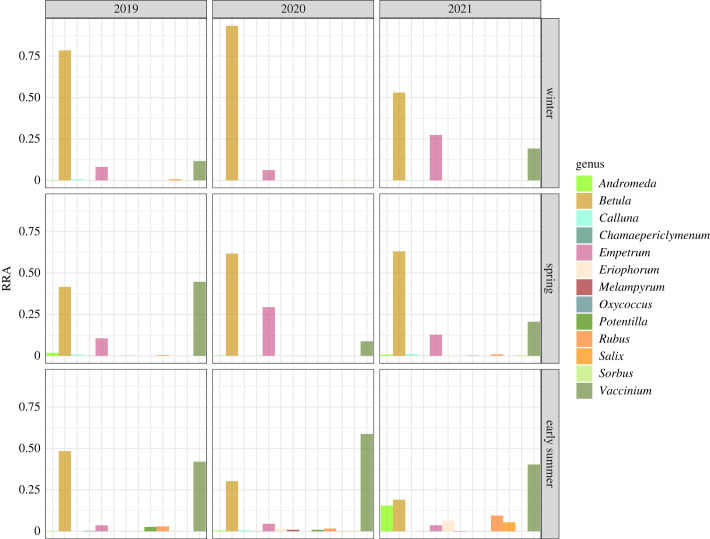
Stacked bar chart showing the proportions of reads assigned to taxonomic range genus from the fecal samples collected from willow ptarmigan (*n* = 99). The samples are distributed over the three sampling-years, and the field period is separated in three periods to represent the development from winter to early summer.

Similar to the FO data, the highest RRA of consumed functional groups were trees and dwarf shrubs. Of the analysed sequences, trees represented as much as 54.7% of the functional groups while dwarf shrub represented 41.8%. Only 1.8% of the total reads originated from the group forbs and 0.8% from graminoids. *Betulaceae* was the most abundant family (54% of total RRA) and was represented primarily by *Betula* sp. (27.1% of total RRA) and *Betula nana* (26.7%). *Ericaceae* was the second most abundant family (41.8%) where *Vaccinium myrtillus* (20.7% of the total RRA) and *Empetrum nigrum* (11.3%) were the ASVs with the highest RRA (figure 2).

From each sample, we identified on average 6.2 ASVs (range: 2–14) at the species level, 2.5 ASVs (range: 1–6) at the family level, and 2.3 functional groups (range: 1–4) per sample. When grouping the field period spanning winter to early summer into three time periods, we identified 12 ASVs (range per sample: 2–9, *n* = 33), 16 ASVs (range per sample: 3–11, *n* = 36) and 18 ASVs (range per sample: 4–14, *n* = 30) at the species level in samples from winter, spring and early summer, respectively.

**Figure 3.**
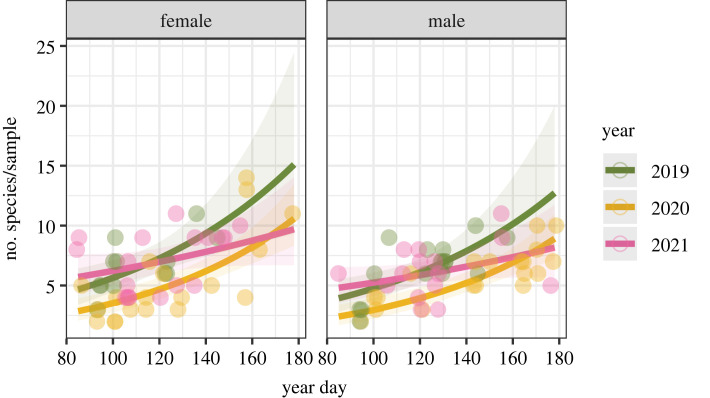
Estimated relationship (solid line) between number of species in samples and day of the year. The shaded ribbons represent 95% confidence interval. Function jitter is used for visualization of data points to avoid overlapping points.

The number of plant species per sample was best described by a model including Julian Date, year and sex and the interaction Julian Date × year (electronic supplementary material, table S3). All other candidate models received substantially less support (ΔAICc > 2.84). Based on this model, the number of species in the willow ptarmigan diet increased with day of the year, but this seasonal progression differed between years (electronic supplementary material, table S4). In addition, females had more diverse diets than males (figure 3). Similar results were found when modelling number of families and number of functional groups as a function of season, year and sex (electronic supplementary material, table S5; table S6).

### 3.2. Seasonal and annual variation in occurrence of the most frequent diet items

**Figure 4.**
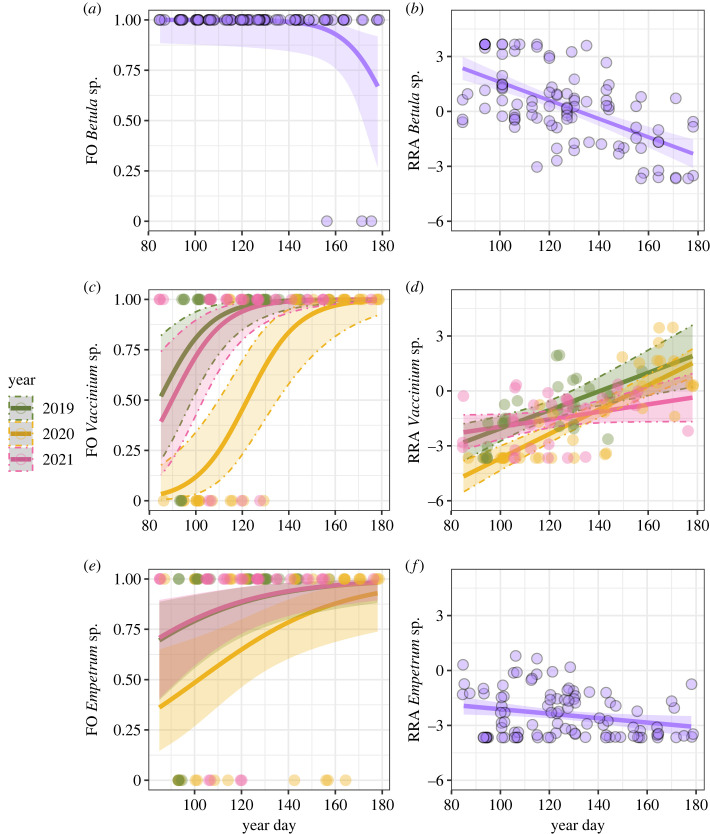
Estimated relationship (solid line) between FO and RRA of *Betula* (*a,b*), *Vaccinium* (*c,d*) and *Empetrum* (*e,f*) in samples and day of the year. The shaded ribbons represent 95% confidence interval. Function jitter is used for visualization of data points to avoid overlapping points.

The FO of *Betula* spp*.* was best described with a model including only Julian Date (electronic supplementary material, tables S7 and S8), showing a decrease in occurrence of this diet item as season progressed (figure 4*a*). Models including NDVI or NDSI received substantially less support in the data (ΔAICc = 6.13 and ΔAICc = 6.62). When shifting the focus to *Betula* spp*.* RRA, three nested models were competitive (electronic supplementary material, tables S9 and S10). The models revealed clear effect of Julian Date on the abundance of *Betula* spp*.* in samples (electronic supplementary material, table S9), whereas the evidence for effects of year or interaction with year was inconclusive (electronic supplementary material, table S9). The relative abundance of *Betula* in the diet decreased with Julian Date (figure 4*b*).

Model selection revealed that a model including the additive effects of Julian Date and Year was most supported by the date: in general, the FO of *Vaccinium* spp*.* in the fecal pellets increased with Julian Date and was consistently higher in 2019 and 2021 compared to 2020 (figure 4*c*; electronic supplementary material, tables S11 and S12). This model had substantially more support than the other candidate models (all models ΔAICc greater than 13.91). When including only the first observation for each bird (see Methods), the models were similar (electronic supplementary material, tables S13 and S14). Moreover, when modelling the RRA of *Vaccinium* spp*.* in the diet as a function of season and year, we found strongest support for the full model including the main effects Julian Date and year, and the interaction between them (electronic supplementary material, tables S15 and S16). All other models received substantially less support (ΔAICc > 7.22). The *Vaccinium* RRA in samples increased with Julian Date, but the increase in the proportion of *Vaccinium* and the relative abundance of *Vaccinium* spp*.* in the diet were different between years (figure 4*d*).

As season progressed, FO of *Empetrum* spp. increased, and in general *Empetrum* spp. was less frequently included in the diet in the year 2020 than in the two other years (electronic supplementary material, tables S17 and S18; figure 4*e*). However, the model including NDSI (ΔAICc = 0.73) and the model including the main effects Julian Date and Year (ΔAICc = 0.88) were competitive, indicating that snow cover may explain the probability of the willow ptarmigan to include *Empetrum* in their diet. When modelling RRA of *Empetrum* spp. as a function of season and year (electronic supplementary material, tables S19 and S20) the first two models were nested and revealed an effect of Julian Date on the abundance of *Empetrum* (model jDate in electronic supplementary material, table S19), whereas the evidence for effects of year or interactions with year was inconclusive (electronic supplementary material, table S20). Owing to higher AICc (ΔAICc < 1.98) and low AICcWt (less than 0.14) the third model was considered to explain only a minor part of the variation in abundance of *Empetrum* in the samples. The relative importance of *Empetrum* in the diet decreased with Julian Date (figure 4*f*).

### 3.3. Community composition

We applied separate perMANOVA tests to examine the effects of sampling period, year, and sex on the dietary composition based on FO and RRA, respectively. Both when considering FO and RRA there were clear indications of differences in the diet composition between fecal samples collected from the sampling periods and years (FO: d.f. = 4, *F* = 2.07 *p* = 0.009; RRA: d.f. = 4, *F* = 3.06, *p* = 0.002). However, there was no consistent evidence for differences in dietary composition between males and females (FO: d.f. = 1, *F* = 1.88 *p* = 0.112; RRA: d.f. = 1, *F* = 1.25, *p* = 0.28).

## 4. Discussion

Based on DNA metabarcoding of fecal samples from willow ptarmigan, we identified 18 important diet components during the transition from winter to early summer. We found that species from the functional groups trees and dwarf shrubs dominated the diet. Within these groups three genera constituted the most important diet components: *Betula, Vaccinium* and *Empetrum.* These genera differed in temporal occurrence and relative abundance throughout the season. Partly in line with our first prediction we found that the winter diet of willow ptarmigan was dominated by *Betula* species, while elements of nutritious field vegetation increased as the spring progressed. While *Betula* constituted a declining part of the diet throughout the spring, the occurrence and abundance of dwarf shrubs, forbs and graminoids increased. However, dwarf shrubs, especially *Empetrum*, also constituted an important part of the winter diet of willow ptarmigan. We found that diet richness and composition varied across time, but in contrast to our second prediction, this dietary variation was explained to a larger extent by Julian Date than by snow cover (NDSI) and vegetation phenology (NDVI). Finally, there was some support for a sex-bias in diet: females had a more diverse diet than males, but we found no support for a difference in diet composition between sexes across all fecal samples.

### 4.1. Temporal variation in key dietary components

Herbivore species living in seasonal environments are expected to make foraging choices that track the timing of spring vegetation growth and the seasonal availability of nutritious plants [46]. For willow ptarmigan, access to nutritious field vegetation in spring is important to acquire energy reserves for the breeding period after a harsh winter [15,47]. A main finding of this study is that willow ptarmigan diet composition and richness shifts gradually during the transition from winter to early summer. As predicted, they switch from a relatively narrow winter diet to broader spring diet with increasing elements of nutritious field vegetation. This is expected given that vegetation is subject to seasonal effects, and is consistent with previous studies [14,15]. During winter, the diet was dominated by *Betula* spp., but as the season progressed the relative abundance of *Betula* spp. declined. Notably, there was no clear difference in *Betula* FO or RRA between years. Annual variations in the arrival of spring and snow cover therefore do not seem to affect willow ptarmigan intake of *Betula,* and this is probably related to the types of food items that are most available during the winter when snow depth is at its highest. Several studies from other areas have pointed out willow (*Salix* spp.) as the preferred winter food for willow ptarmigan [11,15,20]. However, willow is much less abundant than birch in our study area and largely snow-covered during the winter. Brittas [15] found that during winter the annual variation in diet proportions of willow ptarmigan mainly appears to be related to variation in food abundance, and suggested that annual variation in food quality has little effect on willow ptarmigan winter diet composition.

Although a narrow winter diet is documented in previous studies, metabarcoding provides a better taxonomic resolution that give the possibility of a broader identification of species which can be difficult to distinguish from each other under traditional methods [48]. As many as 12 of 18 identified ASVs were also detected in the winter diet, indicating that willow ptarmigan takes advantage of a wider winter diet than previously suggested. This richness is probably due to easier access to last year's plant production in areas where the snow cover is sparse. Whereas Brittas [15], during a 6-year study in Sweden, only detected 0.5% dwarf shrubs (and only blueberries) in late winter diet, we have shown that dwarf shrubs, and especially crowberry, seem to be important elements in the willow ptarmigan winter diet. For example, in 2021 *Empetrum* and *Vaccinium* accounted for over 50% of the total RRA from the samples from winter (figure 2). Crowberry grows on exposed ridges in the mountains, which can often be without snow cover. Willow ptarmigan thus has the opportunity to use both evergreen plant elements and last-season berries from crowberry as a food resource throughout the winter. Along with crowberry, species in the genus *Vaccinium* are also important elements in the winter diet. However, while the abundance of *Empetrum* decreased during the transition to spring, the focus on *Vaccinium* increased. The importance of bilberry shoots as spring food was highlighted by Brittas [15] and Pulliainen & Tunkkari [14] and is also confirmed in this study. However, our findings propose that willow ptarmigan utilize a wider range of *Vaccinium* species in spring than documented in earlier studies.

In spring, plants from the functional group dwarf shrubs are just as abundant in the diet as trees, and in early summer dwarf shrubs dominate the diet along with increasing elements of graminoids and forbs. For all three periods together, only a small percentage of the plants in the diet were represented by forbs and graminoids. This may be attributed to the fact that sampling occurred in late winter and spring, and species from these groups will probably become more abundant over the summer. Although these functional groups were present only in small proportions, previous studies have shown that they are of great importance for the ptarmigan. For example, Brittas [15] revealed that even though cotton grass was a minor component in the diet of willow ptarmigan in spring, a positive correlation between food digestibility and the percentage of cotton grass flowers in the diet was found. The shift in feeding process from winter to early summer is related both to seasonality and diet quality. A reduced spring snow cover affects feeding opportunities for willow ptarmigan, and by shifting from a diet dominated by trees to a broader diet with dwarf shrubs, graminoids and forbs, they obtain a more nutritious diet. This shift in diet results in a spring diet with lower fibre content and a higher ash, fat and protein content than in winter, which in turn increases the nutrient value and digestibility of the diet significantly [15,49]. The importance of fresh plant material in the spring diet of ptarmigan is well documented, and they are found to select the most nutritious food items out of proportion to its availability [21,49]. They also tend to breed better in years with early spring, but it is not clear whether this is due to high digestible-protein content, relatively high digestibility or both [49]. Also in our study spring diet composition and richness varied between years. Since spring diet affects breeding success [15,50], these annual variations in diet are likely to affect willow ptarmigan condition and fitness also in our study area, but this has not yet been investigated in detail.

### 4.2. The variation in diet richness and composition as explained by the abiotic factors vegetation phenology and snow cover

Global warming is expected to affect alpine plant phenology by advancing the timing of plant growth and by prolonging the growing season [3,4]. Owing to higher nutritional values in fresh plant material, alpine herbivore species is expected to make foraging choices that track these changes in plant phenology [46,51]. In our analyses, variations across time in diet composition and diet richness were in general better explained by Julian Date than by snow cover and vegetation phenology measured by NDVI and NDSI, respectively. García-González *et al*. [16] found that the availability and quality of diet items in spring is associated with snowmelt and proposed that snowmelt affects the diet composition of ptarmigan by affecting plant species availability and phenology. However, our results indicate that on a finer temporal scale there may also be other factors that influence willow ptarmigan choice of diet than just availability. Even though NDVI and NDSI give us good indices on the amount of gross primary production and snow cover in the study area, the indices do not necessarily represent the areas where the ptarmigans are found. Willow ptarmigan are heterogeneously distributed across the landscape [8], and within their range individual birds generally prefer habitats with a high density of food and cover from predators [52]. Some factors related to life-history traits of the species might determine its distribution across a landscape during the transition from winter to spring, and this might also affect the diet composition. First, males in spring defend breeding territories of 2–12 ha [53]. After mating with a male in late winter/early spring the female feeds almost solely within the male's territory [54], thus location and size of the territory will affect available diet items for both sexes. Second, foraging strategy during snow melt is probably a trade-off between foraging in patches with high-quality food and cover from predators [19,55]. An earlier snowmelt in the alpine areas can lead to a mismatch between the ptarmigan moulting and the presence of snow [56] and this may cause the ptarmigan to seek areas with snow during foraging even though green biomass is available. Although global warming is assumed to lead to an earlier access to green nutrient-rich plant material, it does not necessarily lead to a temporal change in willow ptarmigan diet composition.

In support of our prediction, we found clear inter-annual variation in diet composition. However, this annual variation was not well explained statistically by either NDVI or NDSI. One reason might be that the composition of plant species in the willow ptarmigan diet show cyclicity and therefore have an annual and seasonal variation in both nutrient content and abundance [15]. Since the willow ptarmigan has the ability to be selective during foraging [57], this cyclicity will affect what the birds choose to eat as well as what they have access to. After snowmelt, alpine plants experience a rapid growth period associated with an increase in nutrients and digestibility [51]. There will be variations between different plant species as to when they reach their peak in nutrition, and in a study of Pyrenean rock ptarmigan (*Lagopus muta pyrenaica*) García-González *et al*. [17] found that diet quality is more dependent on the phenological stage of food components than their floristic composition. The timing of snow melt differs between years, and this affects plant phenology and species peak in nutrition. This, in addition to plant cyclicity, can possibly result in annual variations in willow ptarmigan diet composition. Abundance, digestibility, and nutrition of different diet items are expected to change throughout the season [15,21]. Unfortunately, we do not have reliable data for this subject in our study area. Future studies should investigate the relationship between plant phenology, diet quality, abundance and willow ptarmigan diet further.

### 4.3. Sex variation in willow ptarmigan diet

We did not detect a difference in diet composition between male and female willow ptarmigan. This is in accordance with previous studies from Fennoscandia [14,15]. However, we found that females have a more diverse diet than males. Metabarcoding can give better taxonomic resolution and identify a wider breadth of diet items compared to traditional methods [58], so this result could have been overlooked in studies using classical approaches. In a study from Canada, Elson *et al*. [20] showed that female willow ptarmigan feed on higher nutritive quality foods than males in winter and prior to egg lying. It is conceivable that the difference in diet richness supports the fact that females are more selective and search for plants that contain higher nutritive foods during the spring than males. The detected difference could also be due to sex-specific habitat selection patterns, and further studies are required to answer this question.

### 4.4. Metabarcoding of fecal pellets in dietary studies of herbivore birds

This study demonstrates that metabarcoding of fecal pellets is suited to estimate the dominant food plants in the diet of willow ptarmigan. This non-invasive method is more sensitive to detect a larger variety of diet items than traditional methods as examination of crop material and observation studies [58–60]. Collection of fecal pellets represents a field method that is easy to implement and collecting fecal samples over temporal and spatial scales provides the opportunity to assess factors that affect diet composition. However, it must also be noted that fecal samples provide a snapshot of the bird's diet over the hours or days preceding defecation, and may contain diet items that were consumed at other locations than the area where the feces were collected. This can make it challenging to link environmental parameters to diet on fine spatial scales. Furthermore, different diet items will vary both in their digestibility, and the degree to which their DNA is degraded in the digestive system. This impacts both the probability of detection for any given diet item, as well as contributes to the fact that the relative abundance of a given diet item inferred from feces may not directly correlate with its importance in the bird's energy budget [61]. The molecular methods applied in this study also have some limitations in assessing the diet via analysis of fecal pellets. First, 21 of 141 samples failed to amplify during the PCR amplification process, and this may be related to DNA degradation due to the time between defecation and sampling [36]. This highlights the importance of sampling pellets as fresh as possible. Second, the primers used have some limitations for detecting ASVs in family Pinaceae and Cupressaceae which in theory could include potential forage plants [28]. Further, the characteristics that make metabarcoding highly sensitive to detect species, also make the method vulnerable to potential contamination and detection of false positive elements in the diet [35]. In this study, 27 detected species were found to be unlikely for the study area and deemed contaminants. Contaminant DNA may be introduced into laboratory reagents in production facilities, or could be introduced by human handling of samples in the field or in the laboratory, as evidenced by the detection of common human foods like cucumber and spinach. Alternatively, some of these presumed contaminants may represent sequences that are incorrectly identified due to incomplete reference databases or poor marker performance for select plant taxa. As there is evidence of low level potential for contamination in the dataset, we systematically excluded all species that had the same or less total sequence amount than the contaminant species with the highest total in order to avoid any potential false positives due to cross contamination. This meant that 68 of 86 ASVs were deemed contaminants or false positives and excluded from further analyses and results. The removed species represented a very small proportion of the sequences and thus were considered not to be important dietary components. However, it is likely that some of the species that were removed were in fact true diet items. For example, alpine bistort (*Bistorta vivipara*) which has been reported to be a preferred plant by other species of ptarmigan [62], was removed in this operation. The list of documented food items can thus be considered as a minimum list of their entire diet composition.

## 5. Conclusion

Through this study, we have shown that metabarcoding of fecal pellets is a successful method for gaining insight into the diet of willow ptarmigan, and that this method, in combination with remote sensing tools, can provide the opportunity to investigate how seasonal variables affect the diet over temporal and spatial scales. The willow ptarmigan diet composition and richness were found to vary both seasonally and annually. Snow cover and vegetation phenology, as measured by NDVI and NDSI, respectively, were however not found to adequately explain the variation in diet. This could indicate that although snow cover and vegetation phenology affect the availability and abundance of plant food in ptarmigan habitats, other intrinsic and extrinsic factors can contribute to explaining the temporal variation in ptarmigan diet. However, limitations in use of satellite-based data could also have affected the results, and validation through local methods is therefore needed.

## Data Availability

Data (processed and pre-processed), additional information and code scripts are available from a registered open archive hosted by the Open Science Framework (https://doi.org/10.17605/OSF.IO/ZRQVC) [63]. The sequence data generated and/or analysed during the current study are available in the NCBI Short Read Archive under project accession number PRJNA987594. Additional information is available in electronic supplementary material [64].
